# Prognostic significance of circulating microRNA-214 and -126 in dogs with appendicular osteosarcoma receiving amputation and chemotherapy

**DOI:** 10.1186/s12917-019-1776-1

**Published:** 2019-01-25

**Authors:** Kazuki Heishima, Travis Meuten, Kyoko Yoshida, Takashi Mori, Douglas H. Thamm

**Affiliations:** 1 Laboratory of Veterinary Clinical Oncology, Faculty of Applied Biological Sciences, Gifu University, 1-1 Yanagido, Gifu, Japan; 2 Flint Animal Cancer Center, Department of Clinical Sciences, Colorado State University, Fort Collins, CO 80523 USA; 3 Center for Highly Advanced Integration of Nano and Life Sciences (G-CHAIN), Gifu University, 1-1 Yanagido, Gifu, Japan

**Keywords:** Bone, Cancer, Canine, Prognosis, Biomarker, Comparative oncology

## Abstract

**Background:**

Dogs with appendicular osteosarcoma (OSA) receiving standard amputation and adjuvant chemotherapy demonstrate variable outcome with treatment; however, additional biomarkers would be helpful for predicting their outcome. In the present study, we assessed the potential of circulating microRNA-214 (miR-214) and − 126 (miR-126) to predict time to metastasis and death in dogs with OSA treated with amputation and chemotherapy.

**Results:**

Seventy-six dogs that fully met inclusion criteria were included in the analysis. The criteria included (1) a diagnosis of appendicular OSA without metastases at diagnosis, (2) treatment by amputation and chemotherapy using carboplatin, doxorubicin, cisplatin, or a combination of these agents. Circulating miR-214 and -126 levels at the time before treatment were measured by using RT-qPCR. High circulating miR-214 and serum alkaline phosphatase (ALP) significantly predicted short disease-free survival (DFS) and overall survival (OS). Conversely, high circulating miR-126 significantly predicted prolonged DFS and OS. An integrated approach using circulating miR-214, − 126, and serum ALP showed better accuracy in the prediction of DFS and OS and identification of long-term survivors than prediction using only ALP. Other variables (age, weight, sex, monocyte counts, and primary tumor site) were associated with neither DFS nor OS. miRNA levels did not strongly correlate with histopathological indices.

**Conclusions:**

Circulating miR-214, − 126, and an integrated prognostic score have strong potential to predict the outcome of canine appendicular OSA patients receiving amputation and chemotherapy.

**Electronic supplementary material:**

The online version of this article (10.1186/s12917-019-1776-1) contains supplementary material, which is available to authorized users.

## Background

Appendicular osteosarcoma (OSA) is a lethal neoplastic disease and a major cause of death for large breed dogs. The median survival time (MST) of OSA receiving limb amputation alone is only 5 months. The 1- and 2-year survival rates are 19.8 and 2%, respectively, and very few cases survive for 2 years [1]. Postoperative chemotherapy using doxorubicin, cisplatin, or carboplatin after amputation significantly extends the MST to approximately 12 months with a 45.5% 1-year survival rate and 20.9% 2-year survival rate [2]. Moreover, a certain number of cases show an especially good outcome following chemotherapy and survive for three years or longer [3]. However, a half or more of OSA cases will succumb to metastasis and subsequent death within a year [4, 5]. This implies that OSA contains several populations having different sensitivity to chemotherapy.

OSA cases having a poor outcome following conventional chemotherapy may have a better outcome if they could be identified and receive more aggressive or alternative treatment protocols. However, conventional tissue- or blood-based markers are inaccurate for identifying these cases. OSA shows high intratumoral heterogeneity; i.e., a small biopsy sample may not be representative of the entire lesion [6, 7]. Thus, tissue-based markers using histopathology [8, 9], protein [10–12], and mRNA [13] may be inaccurate in providing precise and consistent prognostication in OSA. Blood-based biomarkers, on the other hand, may capture overall features of such a heterogeneous tumor; therefore, they are more suitable to evaluate the pathobiology of OSA. However, serum alkaline phosphatase (ALP), the only available blood-based marker for OSA [14–16], lacks sufficient accuracy. The activity of ALP can be altered by numerous nonspecific causes including hepatic injury, endogenous or exogenous corticosteroid use, and bone damage [17]. Therefore, novel blood-based biomarkers are necessary for accurately predicting the disease course of OSA receiving standard chemotherapy.

OSA is also a critical disease in humans, and biomarkers that predict prognosis after chemotherapy are in need as well. However, the exploration of novel biomarkers for human OSA had been challenging because human OSA is a rare cancer, and it is difficult to collect sufficient numbers of samples and clinical information [18]. Conversely, samples of canine OSA are more available due to its very high prevalence in large breed dogs [19]. Furthermore, the findings from canine OSA studies are likely to be translatable to human OSA because human and canine OSA have many shared histopathologic and genetic features [20–22]. In addition, spontaneous canine OSA retains tumor microenvironmental features of the human disease, including equivalent histologic and genetic heterogeneity [23]. Therefore, the exploration of candidate biomarkers in canine OSA could also be beneficial for human OSA patients.

MicroRNAs (miRNAs), evolutionally conserved small non-coding RNAs, have a strong association with oncogenesis through post-transcriptionally controlling diverse biological processes including cell proliferation, differentiation, apoptosis, and metastasis [24]. Some of these cancer-associated miRNAs are released and detectable in the bloodstream, and the concentrations in the blood directly reflect the pathologic state of the tumor [25, 26]. These circulating miRNAs are stable and readily measurable by using conventional reverse transcription quantitative polymerase chain reaction (RT-qPCR) techniques [26]. Furthermore, circulating miRNAs may capture comprehensive features of histologically heterogeneous tumors such as OSA, because the circulating levels are the average of the total miRNAs secreted from the tumor tissue.

MicroRNA-214 (miR-214) and microRNA-126 (miR-126) have shown strong associations with the pathogenesis of human OSA. miR-214 and -126 regulate the proliferation, survival, metastasis, and chemoresistance of human OSA [27–36], and their tissue expression has shown prognostic potential in the estimation of metastasis and death [37, 38]. Furthermore, miR-214 and -126 are likely to be associated with the pathogenesis of canine OSA. The sequences of these miRNAs are identical between human and canine. In addition, our previous study revealed that dogs bearing OSA demonstrate aberrantly high plasma levels of these two miRNAs [39]. These findings suggest that miR-214 and -126 are factors that play essential roles in both human and canine OSA. Therefore, we hypothesized that circulating miR-214 and -126 may serve as low-invasive, specific, and sensitive prognostic biomarkers for both human and canine appendicular OSA receiving chemotherapy.

The purpose of the present study was to assess the potential of circulating miR-214 and -126 to predict the disease course and the treatment response of appendicular OSA receiving amputation and adjuvant chemotherapy.

## Results

### Cohort characteristics

**Fig. 1:**
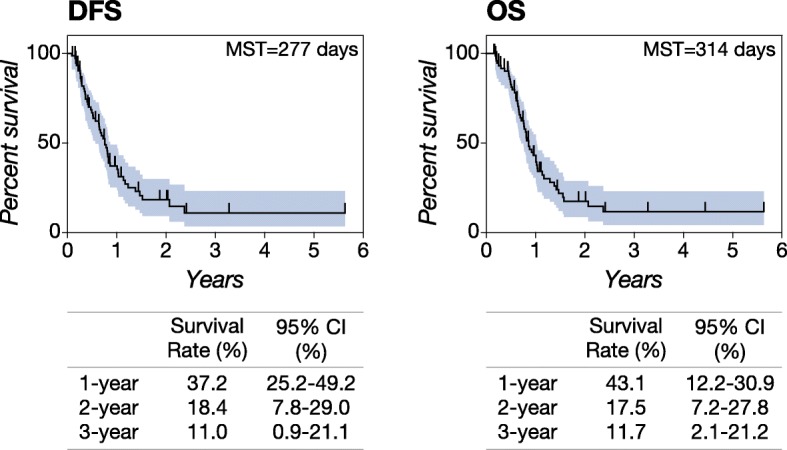
Disease-free survival and overall survival for the entire cohort. The cohort represented typical prognosis of dogs bearing appendicular osteosarcoma. Kaplan-Meier (KM) curves showing the disease-free survival (DFS) and the overall survival (OS) times of the cohort. The events and censored counts in the cohort were 52 and 24, respectively

We evaluated a total of 106 plasma samples of appendicular OSA in the present study. The 106 cases were used for the assessment of circulating miR-214 and -126 profiles, and 76 cases that fully met the criteria were included in the subsequent survival analysis (Additional file 1: Figure S1a). The cohort is consistent with other cohorts of dogs bearing appendicular OSA and receiving standard therapy (Additional file 1: Figure S1b, Table 1). The typical characteristics were old, neutered, and large breed dogs. The median age and weight of the cohort were 8.2 years old and 36 kg, respectively. A majority of cases (71%, 54 of 76 cases) had primary sites in the forelimb (humerus, radius, and ulna), and the most common primary site was the radius (38.2%, 29 of 76 cases). The cohort contained several different prognostic groups (Fig. 1). A majority of the population (approximately 56–63%) showed either or both metastasis and death by one year after diagnosis despite treatment (low response group). Approximately 19–29% cases survived for more than a year but less than two years (medium response group). Most of the rest that survived for two years (approximately 11%) remained metastasis-free and alive long-term (high response group). The median disease-free survival (DFS) and overall survival (OS) times of the entire population were 277 and 314 days respectively, and the 2-year disease free/overall survival percentage was approximately 18%. The type of postoperative chemotherapy utilized did not have a significant effect on DFS or OS (Additional file 1: Figure S2).

**Table 1:** Characteristics of the cohort for survival analysis (primary sites, sex, and breeds)

	Case Number	%
Primary sites		
Radius	29	38.2
Humerus	24	31.6
Tibia	10	13.2
Femur	12	15.8
Ulna	1	1.3
Sex		
FS	38	50.0
MC	35	46.1
F	1	1.3
M	2	2.6
Breeds		
Mix	15	19.2
Labrador Retriever	13	16.7
Golden Retriever	8	10.3
Great Dane	4	5.1
Rottweiler	4	5.1
American Staffordshire Terrier	3	3.8
English Bulldog	3	3.8
Greyhound	3	3.8
Akita	2	2.6
Australian Cattle Dog	2	2.6
Bernese Mountain Dog	2	2.6
Great Pyrenees	2	2.6
Irish Setter	2	2.6
Mastiff	2	2.6
Saint Bernard	2	2.6
Anatolian Shepherd	1	1.3
Bullmastiff	1	1.3
Coonhound	1	1.3
Doberman Pinscher	1	1.3
Flat-Coated Retriever	1	1.3
German Shepherd Dog	1	1.3
German Wirehaired Pointer	1	1.3
Norwegian Elkhound	1	1.3
Old English Sheepdog	1	1.3
Rhodesian Ridgeback	1	1.3
Swiss Mountain Dog	1	1.3

### Independent profiles of circulating miR-214 and -126 in appendicular canine OSA

We performed miRNA RT-qPCR to obtain the profiles of circulating miR-214 and -126 in appendicular OSA. We first normalized the raw relative quantities (RQ) to calibrated relative quantities (CRQ) by using the inter-assay control [40]. In the preliminary analysis, we identified bias of miRNA levels due to different conditions of hemolysis and the anticoagulants used (ethylenediaminetetraacetic acid, EDTA or sodium citrate, SC). The hemolyzed and EDTA-anticoagulated samples showed relatively low CRQ for miR-214 and -126 (Additional file 1: Figure S3a, b, c, e). We also experimentally demonstrated that similar changes occurred in hemolyzed and EDTA-anticoagulated samples (Additional file 1: Figure S4a, b, c, d). Therefore, we normalized the CRQ by using the median of the hemolytic grades and anticoagulant types (Additional file 1: Figure S3d, e). The normalized CRQ, adjusted-CRQ (ACRQ), was used in the subsequent analysis to quantify circulating miR-214 and -126 levels. We also calculated the normalized -∆Ct values as a reference (Additional file 1: Figure S5a).

We then evaluated the profiles of circulating miR-214 and miR-126 in the cohort. The levels of both miRNAs showed normal distribution after log-transformation (Additional file 1: Figure S6). Hierarchical cluster analysis and scatter plots showed that circulating miR-214 and -126 had distinct patterns of profiles with no significant linear correlation (Pearson correlation, *P* = 0.1, Additional file 1: Figure S7).

### Minimal association of circulating miR-214 and -126 profiles with histopathologic index

We examined the association between circulating miRNA profiles and histopathologic indices (histopathological grades, mitotic index, and histological subtypes) in the cases for which hematoxylin and eosin (H&E) slides were available (*n* = 34). Circulating miR-214 and -126 showed no association with most histopathologic indices (Additional file 1: Figure S8a, b), but some association with histopathological grade. Using the Kirpensteijn system, circulating miR-214 levels were high in Grade III compared to Grade II tumors. Using the Straw/Powers system, circulating miR-126 levels were high in Grade III compared to Grade II tumors. There was no correlation between circulating miR-214 and -126 and mitotic index (Additional file 1: Figure S8b). Most cases were the osteoblastic subtype, and there were very few of other histologic subtypes (Additional file 1: Figure S8c). The survival difference between histopathologic grades was not evaluated due to an insufficient number of cases having both a histopathologic section for review and survival data (*n* = 4).

### Univariate prognostic potential of circulating miR-214, − 126, and other clinical parameters

**Fig. 2:**
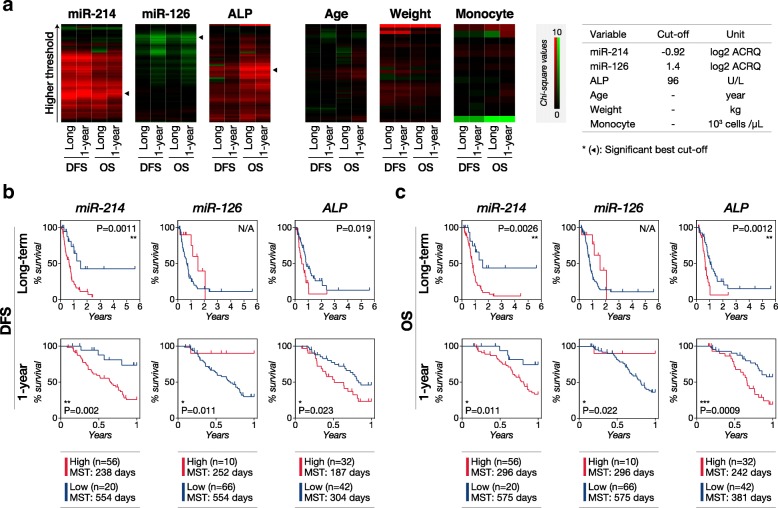
Univariate prognostic potential of circulating miR-214, − 126, and other variables. Circulating miR-214, − 126, and serum ALP showed significant association with short disease-free survival (DFS) and overall survival (OS), although circulating miR-126 was prognostic only in the 1-year analysis. Circulating miR-214 discriminated the long-term survivors well while serum ALP did not. **(a)** X-tile analysis for determining the best cut-off values of the variables. The arrowheads indicate the best cut-off point for each variable. The red and green in the heatmap indicate high chi-square values toward good and poor prognosis, respectively. Kaplan-Meier curves indicating **(b)** DFS and **(c)** OS with the separation by the best-cut off for miR-214, miR-126, and serum ALP in long-term and 1-year analysis. *P*-values were calculated by using the log-rank test. P-values less than 0.05 were considered to be significant

To identify the variables that were potentially associated with DFS and OS, we performed univariate Cox regression analysis (Table 2). The variables included were age, weight, sex, primary sites, serum ALP, monocyte count, circulating miR-214, and − 126. We first determined the best cut-off for each continuous variable by using X-tile analysis. X-tile analysis optimized cut-off points for circulating miR-214, − 126, and serum ALP in predicting either long-term (up to six years) or 1-year outcomes (Fig. 2a). Circulating miR-214, miR-126, and serum ALP were predictors of the DFS and OS in OSA patients receiving amputation and chemotherapy (Fig. 2b, c). High circulating miR-214 was significantly associated with short DFS and OS in the both long-term and 1-year analysis (cut-off at − 0.92 of log_2_ ACRQ, 0.48 SD lower point than mean). Furthermore, miR-214 successfully identified most long-term survivors (high response group). High miR-126 levels were significantly associated with prolonged DFS and OS in the 1-year analysis (cut-off at 1.4 of log_2_ ACRQ, 1.11 SD higher point than the mean). However, miR-126 failed to classify the long-term survivors effectively. High serum ALP showed a significant association with short DFS and OS in the both long-term and 1-year analysis (cut-off at 96 U/L). However, serum ALP did not discriminate long-term survivors well. The other variables (age, weight, sex, primary site, and monocyte count) showed no significant association with DFS or OS (Fig. 2a, Table 2).

**Table 2:** Univariate Cox regression analysis

		Long-term DFS			1-year DFS			Long-term OS			1-year OS		
		*P-value*	*HR*	*95%CI*	*P-value*	*HR*	*95%CI*	*P-value*	*HR*	*95%CI*	*P-value*	*HR*	*95%CI*
Age (year)	*8<*	0.48	1.21	0.70–2.11	0.47	1.25	0.68–2.33	0.28	1.36	0.78–2.35	0.12	1.67	0.87–3.26
	*< 8*	0.48	0.82	0.47–1.42	0.47	0.8	0.43–1.48	0.28	0.74	0.43–1.28	0.12	0.6	0.31–1.14
Weight (kg)	*36<*	0.23	1.41	0.80–2.46	0.34	1.37	0.72–2.57	0.37	1.29	0.73–2.26	0.62	1.18	0.60–2.30
	*< 36*	0.23	0.71	0.41–1.25	0.34	0.73	0.39–1.39	0.37	0.78	0.44–1.37	0.62	0.85	0.43–1.66
Sex	*F and SF*	0.49	1.21	0.70–2.11	0.58	0.84	0.45–1.57	0.71	0.9	0.52–1.57	0.76	0.9	0.47–1.75
	*M and CM*	0.49	0.82	0.47–1.43	0.58	1.19	0.64–2.20	0.71	1.11	0.64–1.92	0.76	1.11	0.57–2.11
Primary site	*Femur*	0.81	0.91	0.37–1.90	0.41	0.69	0.24–1.60	0.83	0.91	0.35–1.99	0.27	0.54	0.13–1.52
	*Humerus*	0.3	1.36	0.76–2.38	0.13	1.65	0.86–3.07	0.38	1.29	0.72–2.25	0.32	1.41	0.71–2.71
	*Radius*	0.45	0.81	0.45–1.40	0.56	0.83	0.43–1.55	0.5	0.82	0.46–1.44	0.98	1	0.51–1.93
	*Tibia*	0.92	1.04	0.43–2.16	0.87	0.93	0.32–2.16	0.96	1.02	0.44–2.05	0.8	0.89	0.30–2.08
	*Ulna*	–	–	–	–	–	–	–	–	–	–	–	–
Serum ALP (U/L)	*High (96<)*	0.024 *	1.97	1.10–3.50	0.028 *	2.03	1.08–3.82	0.0021 **	2.54	1.41–4.56	0.0015 **	2.91	1.51–5.73
	*Low (< 96)*	0.024 *	0.51	0.29–0.91	0.028 *	0.49	0.26–0.92	0.0021 **	0.39	0.22–0.71	0.0015 **	0.34	0.17–0.66
Monocyte (10^3^ cells/μL)	*High (0.3<)*	0.66	1.13	0.65–1.98	1	1	0.54–1.85	0.39	1.27	0.73–2.22	0.38	1.33	0.70–2.58
	*Low (< 0.3)*	0.66	0.88	0.51–1.54	1	1	0.54–1.86	0.39	0.79	0.45–1.36	0.38	0.75	0.39–1.43
miR-214 (log_2_ ACRQ)	*High (−0.92<)*	0.0004 ***	3.51	1.68–8.55	0.0007 ***	4.42	1.77–14.8	0.0012 **	3.2	1.53–7.81	0.0054 **	3.53	1.40–11.84
	*Low (<−0.92)*	0.0004 ***	0.29	0.12–0.60	0.0007 ***	0.23	0.067–0.57	0.0012 **	0.31	0.13–0.65	0.0054 **	0.28	0.084–0.71
miR-126 (log_2_ ACRQ)	*High (1.4<)*	–	–	–	0.002 **	0.17	0.0066–0.54	–	–	–	0.0059 **	0.14	0.0077–0.64
	*Low (< 1.4)*	–	–	–	0.002 **	8.6	1.87–152.48	–	–	–	0.0059 **	7.28	1.57–129.33

Circulating miR-214, miR-126, and serum ALP showed significant association with short disease-free survival (DFS) and overall survival (OS) in either or both the long-term and 1-year analysis, while the other parameters showed no significant association with the survivals. The statistical analysis for “ulna” in the primary site section was not performed due to the insufficient case number for statistical analysis. The best cut-off values were determined by using X-tile analysis. If the X-tile analysis failed to identify significant cut-off values (*P*-values less than 0.1), the median was employed as the cut-off value. HR, hazard ratio; 95% CI, 95% confidential interval. *P < 0.05, **P < 0.01, ***P < 0.001

### Development of integrated grading system using miR-214, − 126, and serum ALP

To accomplish more accurate prognostic prediction, we constructed an integrated prognostic grading system using circulating miR-214, − 126, and serum ALP by using multivariate Cox regression analysis. To avoid multicollinearity in the multivariate analysis, we first confirmed that miRNA levels and the other parameters showed no strong correlation (Additional file 1: Figure S9a, b). Although the analysis identified significant correlation between several variables, such as miR-214 and serum ALP, the R^2^ values of these correlations were small (less than 0.2).

We then performed stepwise Cox regression analysis for selecting the optimal variables to predict DFS and OS (Table 3). Circulating miR-214 was the only significant and independent predictor of long-term DFS. On the other hand, the combination of circulating miR-214 and -126 was the best predictor for the 1-year DFS. For OS, the combination of circulating miR-214 and serum ALP showed the best prediction of both long-term and 1-year survival.

**Table 3:** Stepwise multivariate Cox regression analysis

	Long-term DFS			1-year DFS			Long-term OS			1-year OS		
	*P-value*	*HR*	*95%CI*	*P-value*	*HR*	*95%CI*	*P-value*	*HR*	*95%CI*	*P-value*	*HR*	*95%CI*
miR-214 High (ACRQ, 0.92<)	0.0004 ***	3.51	1.68–8.55	0.001 ***	4.24	1.69–14.21	0.005 **	2.95	1.35–7.77	0.013 *	3.17	1.25–10.71
miR-126 Low (ACRQ, < 1.4)	–	–	–	0.003 **	8.09	1.75–143.59	–	–	–	–	–	–
ALP High (U/L, 96<)	–	–	–	–	–	–	0.007 **	2.26	1.26–4.06	0.0047 **	2.6	1.34–5.15
Model fitness	0.0004 ***	–	–	< 0.0001 ***	–	–	0.0002 ***	–	–	0.0003 ***	–	–

Stepwise multivariate Cox regression analysis identified independent variables for the prediction of the DFS and OS. P-values less than 0.05 were considered to be significant (*P < 0.05, **P < 0.01, ***P < 0.001). HR, hazard ratio; 95% CI, 95% confidential interval

We then constructed an integrated grading system based on the hazard ratios calculated by multivariate Cox regression (Table 4). We considered all variables to have the same impact on the outcome in the grading system because these variables had similar hazard ratios. We also evaluated ALP as a sole prognostic factor. The single ALP grades were separated at the threshold of 96 U/L (the best cut-off determined by the X-tile analysis).

**Table 4:** Definition of the integrated and single ALP grades

		Integrated grades		ALP grades	
		*Criteria*	*Grades*	*Criteria*	*Grades*
DFS prediction	*long-term*	miR-214 > −0.92	G1: Negative G2: Positive	ALP > 96	G1: Negative G2: Positive
	*1-year*	miR-214 > − 0.92 miR-126 < 1.4	G1: Negative G2: 1 positive G3: 2 positives	ALP > 96	G1: Negative G2: Positive
OS prediction	*long-term & 1-year*	miR-214 > − 0.92 ALP > 96	G1: Negative G2: 1 positive G3: 2 positives	ALP > 96	G1: Negative G2: Positive

miR-214 and -126 (log2-transformed ACRQ), ALP (U/L)

**Fig. 3:**
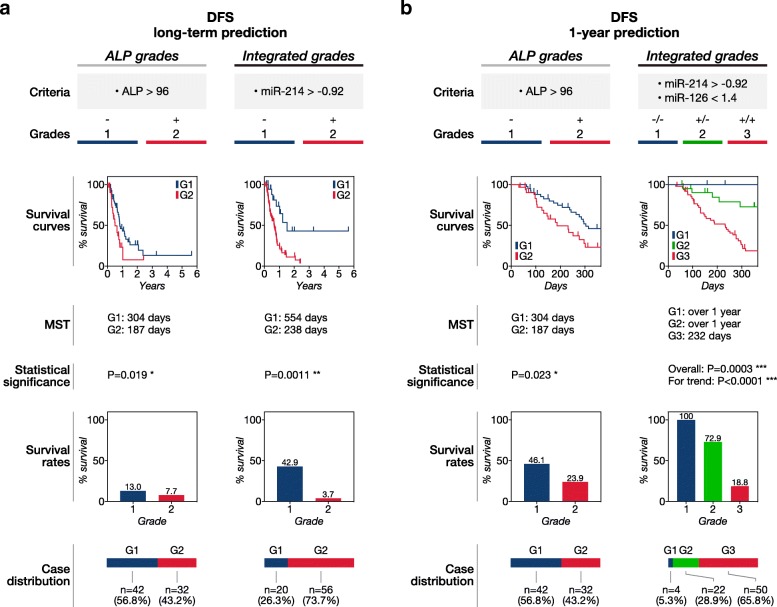
Disease-free survival prediction using the integrated grading system. The integrated grading system using miR-214, − 126, and serum ALP showed more accurate disease-free survival (DFS) prediction than the single ALP grading system. Grading criteria, Kaplan-Meier (KM) survival curves, median survival time (MST), statistical significance, survival rates, and case distribution of the integrated and single ALP grades in the prediction of **(a)** long-term and **(b)** 1-year DFS. Grade 1, 2, and 3 were indicated as G1, G2, and G3 in this figure, respectively. *P*-values were calculated by using the log-rank test or the log-rank test for trend. A P-value less than 0.05 was considered as significant (*P < 0.05, **P < 0.01, ***P < 0.001)

**Fig. 4:**
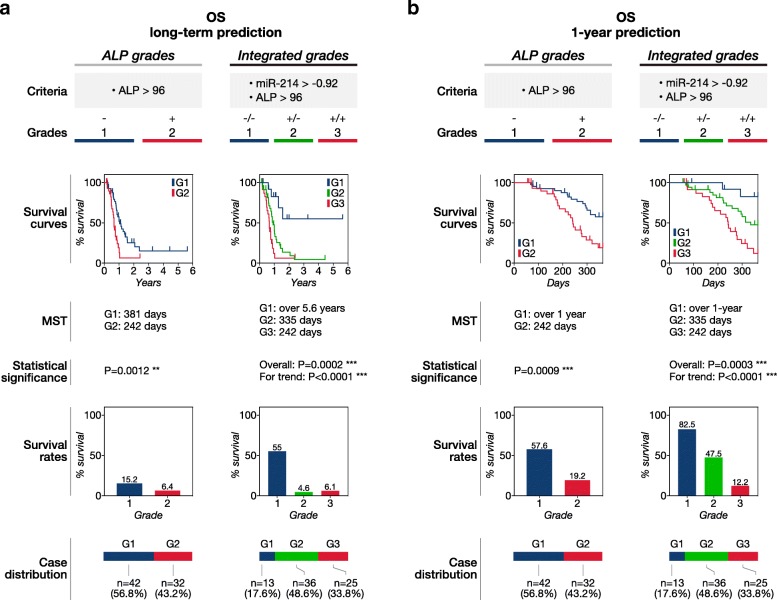
Overall survival prediction using the integrated grading system. The integrated grading system using miR-214, − 126, and serum ALP showed more accurate overall survival (OS) prediction than the single ALP grading system. Grading criteria, Kaplan-Meier (KM) survival curves, median survival time (MST), statistical significance, survival rates, and case distribution of the integrated and single ALP grades in the prediction of **(a)** long-term and **(b)** 1-year DFS. Grade 1, 2, and 3 were indicated as G1, G2, and G3 in this figure, respectively. P-values were calculated by using the log-rank test or the log-rank test for trend. A P-value less than 0.05 was considered as significant (*P < 0.05, **P < 0.01, ***P < 0.001)

We next assessed the long-term and 1-year prognostic potential of the integrated grading system. The integrated grading system showed better classification for predicting survival time than the single ALP grades. (Fig. 3a, b**,** Fig. 4a, b). The integrated grading system showed better separation of Kaplan-Meier (KM) curves in both DFS and OS prediction. The grades via the integrated grading system showed more distinctly separated median survival time (MST) and survival rates than the single ALP grades. Furthermore, the integrated grading system classified most of the long-term metastasis-free and survival cases as Grade 1 while the single ALP grades failed to identify them. Taken together, these results indicated that the integrated grades more accurately predicted DFS and OS of appendicular canine OSA receiving standard therapy than ALP alone.

## Discussion

Here, we demonstrated that levels of circulating miR-214 and -126 before treatment could predict time to metastasis and death after amputation and chemotherapy in dogs with appendicular OSA. Furthermore, the combined use of circulating miR-214, − 126, and serum ALP increased the accuracy of the prediction compared with the sole use of each miRNA or serum ALP. These two circulating miRNAs may serve as non-invasive and specific biomarkers for predicting outcome in dogs with OSA receiving chemotherapy.

Circulating miR-214 and -126, as well as the integrated grading system, may provide several benefits. First, these miRNAs may facilitate to identify the poor prognostic cases resistant to conventional therapy. This may improve understanding of the underlying pathobiology in these cases, and ultimately might contribute to the development of novel therapeutic strategies. Second, these markers may be useful to identify a population that potentially has long-term survival after treatment. The information may reduce unnecessary euthanasia for dogs bearing OSA with a good prognosis, and/or alter some owners’ treatment decisions. Third, these miRNAs might be predictive biomarkers for therapies targeting molecules regulated by these miRNAs when the functional roles and targets of miR-214 and -126 in canine OSA are elucidated.

In veterinary medicine, miR-214 and -126 were originally identified as dysregulated miRNAs in canine hemangiosarcoma [41, 42]. We then demonstrated that these circulating miRNAs are increased in a broad spectrum of canine cancers, and among these, OSA showed extremely high levels of circulating miR-214 and -126 [39]. Although these previous studies suggested the potential of these miRNAs as cancer biomarkers, their prognostic value was not assessed. In the present study, we demonstrated that these circulating miRNAs are significantly associated with time to metastasis and death in appendicular OSA receiving standard of care therapy. Moreover, given that other multiple canine cancers showed an increase of these same miRNAs, circulating miR-214 and -126 may have prognostic potential in various other types of cancers, such as hemangiosarcoma, histiocytic sarcoma, and carcinomas.

In long-term analysis, only circulating miR-214 successfully discriminated most cases having long DFS and OS. Circulating miR-126 wrongly classified the long-term survivors as cases having a poor prognosis. Serum ALP also failed to identify the long-term survivors and randomly classified the long-term survivors as the cases having a good or poor prognosis. The difference between miR-214 and others may be due to the different degree of association with tumorigenesis. miR-214 not only accelerates an aggressive tumor phenotype but also inhibits the differentiation of osteoblasts [43, 44]. However, miR-126 does not affect the regulation of osteoblast differentiation [45], whereas it regulates the tumor activities of OSA. Serum ALP activity could be non-specifically altered by various concurrent diseases, such as hepatic injury, endocrine disease, and bone damage [17]. These previous findings and our results suggest that miR-214 has a strong association with the pivotal regulation of OSA tumorigenesis, whereas circulating miR-126 and serum ALP may have less association, or may indirectly reflect tumor activities.

The conventional prognostic markers previously reported, except for serum ALP, may not be ideal for estimating the DFS and OS of appendicular OSA receiving standard therapy. In the current study, age, weight, and monocyte count showed no significant association with DFS and OS. This result suggests that these markers using signalment and clinical findings are inconsistent and easily biased by many random factors since they do not directly reflect tumor-intrinsic properties. Also, humeral location was not associated with outcome in the current study, although it was a significant prognostic factor in a recent large-scale meta-analysis [46]. This disagreement between the two studies may be due to the differences of the populations included. The previous meta-analysis included cases having a broad range of primary sites including axial skeletal OSA, which typically have a better prognosis than appendicular OSA [9].

Circulating miR-214 and -126 showed no consistent association with histopathological features, although we found some association between circulating miRNAs and histopathological grades: higher levels of circulating miR-214 were found in Kirpensteijn Grade III versus Grade II tumors, and higher levels of circulating miR-126 were found in Straw/Powers Grade III versus Grade II tumors. Also, neither circulating miR-214 nor − 126 levels correlated with mitotic index. Although the result that Kirpensteijn Grade III tumors had higher miR-214 levels than the Grade II partially agrees with the previous report showing that miR-214 was associated with poor prognosis in human OSA, it also displayed inconsistency that the Straw/Powers system categorized the cases having high levels of a favorable prognostic predictor (miR-126) into the high grade. These inconsistent results agree with a previous report showing that histopathological grading failed to predict outcome in dogs receiving standard of care therapy [47].

Several reports suggest that both miR-214 and -126 have strong association with the pathogenesis of human OSA. miR-214 promotes the growth, invasion, metastasis, survival, and chemoresistance of human OSA [27–30, 36]. Conversely, miR-126 inhibits the proliferation, migration, invasion, epithelial-mesenchymal transition, and chemoresistance of human OSA [31–35]. Furthermore, high tissue expression of miR-214 and -126 were associated with short and long survival times in human OSA, respectively [37, 38]. In the current study, both circulating miR-214 and miR-126 showed significant association with DFS and OS. Furthermore, high circulating miR-214 was a predictor of poor prognosis, while high circulating miR-126 was a predictor of favorable prognosis. These consistent results suggest that the function and regulation of miR-214 and -126 are similar between human and dog, and that they may play essential roles in the pathogenesis of canine OSA as in the human homolog.

The prognostic potential and utility of circulating miR-214 and -126 in human OSA have yet to be completely understood. One report suggested potential prognostic value of circulating miR-214; however, the study analyzed survival only in OSA patients already having metastasis, and the case numbers were limited to 20 [48]. For circulating miR-126, there are no reports of the prognostic potential in human OSA. In this study, we found that both circulating miR-214 and -126 have prognostic value in appendicular canine OSA receiving standard therapy. Given that human and canine OSA share many similarities and identical sequences of miR-214 and -126 [20–22, 39], these results suggest that circulating miR-214 and -126 may also predict outcome in metastasis-free human OSA patients.

We acknowledge several limitations in the current study. First, although we clarified circulating profiles of miR-214 and -126 in canine appendicular OSA, the actual source of the circulating miRNAs is still unknown. However, given that the circulating miRNAs showed significant prognostic value, our results strongly suggest that these miRNAs originated from tumor cells and/or tumor microenvironment. Also, regardless of where the miRNAs originate from, these miRNAs are still useful as prognostic markers for canine OSA receiving standard of care. Second, further prospective studies would be necessary for confirming the actual clinical utility and significance of the miRNAs since the design of the current study is retrospective. However, this cohort represented typical characteristics and a typical disease course for canine OSA. Furthermore, the current study only included appendicular OSA cases receiving amputation and standard adjuvant chemotherapy so that potential bias could be minimized. Third, the thresholds of miR-214 and -126 in the current study are not compatible in different experimental conditions because they are relative quantities measured by the qPCR technique. However, the thresholds in the different conditions may be able to be estimated by analyzing small populations. The log-transformed miR-214 and -126 values showed normal distribution, and the distribution in each different condition can be estimated even from small case populations. Therefore, the threshold of miRNAs in each condition can be estimated by using the distance from the mean, which was shown in the current study. Finally, we cannot rule out the possibility that hemolysis still somewhat impacted the accuracy of the prognostic prediction, although we corrected for hemolytic bias. Currently, there is no validated method for correcting miRNA levels affected by hemolysis in dogs. However, several markers are proposed for calibration of miRNAs in human hemolytic blood [49, 50]. The combination use of these hemolysis markers could further improve the accuracy of the prognostic prediction using these miRNAs after the validation in dogs.

## Conclusions

We have demonstrated that circulating miR-214 and -126 have the potential to be predictive markers for predicting metastasis and death of appendicular canine OSA patients receiving amputation and chemotherapy. Also, these results may provide insights for identifying further potential of circulating miR-214 and -126 in human OSA.

## Methods

### Study design

This was a retrospective cohort study. Canine appendicular OSA cases with preserved plasma samples were retrospectively searched from the tumor sample archives at the Flint Animal Cancer Center at Colorado State University (CSU) and used for the assessment of miRNA profiles. The inclusion criteria included (1) a diagnosis of appendicular OSA without metastases at diagnosis determined by 3-view thoracic radiographs with or without additional imaging; (2) treatment by amputation; (3) treatment with at least one dose of injectable chemotherapy using carboplatin (target dose 300 mg/m^2^), doxorubicin (target dose 30 mg/m^2^), cisplatin (target dose 70 mg/m^2^), or a combination of these agents. Cases with an axial location, such as rib, pelvis, head and spine, or those treated with stereotactic radiation therapy or surgical limb sparing procedures (cortical allograft or metal spacer implant) were also excluded.

### Collection of plasma samples

Plasma samples were identified from the tumor archive at CSU. These plasma samples were anticoagulated with either EDTA or SC and had been stored at − 80 °C. Blood samples were collected with informed owner consent and with approval of the Colorado State University Institutional Animal Care and Use Committee.

Plasma samples for inter-assay control of qPCR were obtained from two blood-donor dogs at CSU. Five mL of fresh plasma was taken from these dogs and mixed.

Plasma samples for the hemolysis and anticoagulant experiment were obtained from three blood-donor dogs at Gifu University. For the hemolysis experiment, 10 mL of EDTA-anticoagulated whole blood was carefully taken from these dogs to minimize hemolysis. The 10 mL samples were separated into 3 mL each. The two samples were hemolyzed by repeatedly passing through a 20-gauge needle fitted to a syringe. The samples were centrifuged at 3000 rpm and stored at − 80 °C. For the anticoagulant experiment, 10 mL of whole blood was carefully taken from these dogs to minimize hemolysis. The 10 mL samples were separated into 5 mL each. Half were anticoagulated with EDTA, and the other half with sodium citrate. The samples were centrifuged at 3000 rpm. The samples were stored at − 80 °C. All procedures in obtaining samples were performed in accordance with the relevant guidelines and regulations.

### Collection of clinical and histopathological information

Clinical information was collected from the database at CSU. The information included age, sex, weight, breed, monocyte count, platelet count (Plat.), and serum ALP. Histopathological information including Kirpensteijn grades [8], Straw/Powers grades [9], mitotic index, and histological subtypes was provided via slide review by a single pathologist (TM). The mitotic index was calculated in 0.7 mm^2^ and 2.37 mm^2^ fields because the microscope used in the current study has narrower fields than the common microscope system. The 0.7 mm^2^ and 2.37 mm^2^ fields are equivalent to 3 and 10 high-power fields in the common microscope system, respectively.

### Extraction of total circulating miRNA from plasma

Total circulating RNA was extracted from plasma samples according to a previous report [39]. The NucleoSpin® miRNA Plasma kit (MACHEREY-NAGEL, Düren, Germany) was used for extraction of total circulating RNA. Three hundred μL plasma samples were centrifuged for 3 min at 11,000 x g to remove residual cell debris. Blood proteins were precipitated and removed by using a reagent in the kit. After adjustment of the binding conditions with isopropanol, circulating RNAs were bound to an miRNA collection column. The miRNA was then eluted into 10 μL of RNase-free water. The yield of circulating miRNA from plasma was normalized by using the same amount of plasma sample (300 μL) in each extraction. The purified miRNA was directly and immediately used for the following RT process to prevent degradation of RNA before the PCR step. cDNA was amplified immediately after the RT reaction had been finished to minimize the degradation before the PCR reaction. The RNA quality was assessed by measuring circulating miR-16 levels in the following PCR process [39].

### miRNA reverse transcription quantitative polymerase chain reaction analysis

An miRNA RT-qPCR was performed for measuring levels of circulating miR-214, − 126, and − 16. For the accurate detection and quantification of these short miRNAs having only 22 nucleotides, we performed a looped-primer RT-qPCR using the TaqMan® MicroRNA Assays (Applied Biosystems®, Thermo Fisher Scientific, MA, USA) for miR-214 (AB Assay ID 002306), miR-126 (AB Assay ID 002228) and miR-16 (AB Assay ID 000391) with the TaqMan® MicroRNA Reverse Transcription Kit (Applied Biosystems®). miR-214, − 126, and − 16 were individually reverse-transcribed to cDNA by using the looped-RT primers that specifically reverse-transcribes each miRNA and add extensional sequences for the following PCR amplification. Each cDNA replicate was made in single. The RT condition was as follows. Step 1: 4 °C for 3 min; Step 2: 16 °C for 30 min; Step 3: 42 °C for 30 min; Step 4: 85 °C for 5 min followed by a 4 °C hold. The cDNA unique to each miRNA was subsequently amplified by using pre-designed forward and reverse PCR primers and TaqMan minor-groove-binder probes. These primers specifically bind to cDNA containing the sequences of each miRNA with the extension by the looped-RT primer. cDNA was diluted to 10 times at the final concentration with TaqMan™ Universal Master Mix II (Cat. 4,440,039, Applied Biosystems) and DNase/RNase-free distilled water. The cDNA was amplified with the measurement of fluorescence using a MX3000P qPCR instrument (Stratagene, La Jolla, CA, USA). The cycle conditions used in the PCR step was as follows. Step 1: 50 °C for 2 min; Step 2: 95 °C for 10 min; Step 3: 95 °C for 15 s; Step 4: 60 °C for 1 min; Repeat Steps 3–4 for 50 cycles. All PCR reactions were performed in duplicate. The amplification efficiency of the qPCR reaction was examined and confirmed to be high (96–100%), while the efficiency was not evaluated in all runs or samples.

The threshold cycle (Ct) was calculated by the crossing point method [51]. We used inter-assay control to minimize the errors due to variation of a manual threshold determination and differences of background fluorescence in the samples and runs [40].

miR-16 was the best internal control available for normalizing plasma miRNAs according to our previous study [39] and was selected as an internal control to maintain the consistency with the previous study [39].

### Survival analysis

Survival information including DFS and OS time was retrospectively collected from the medical records. The DFS was defined as the time between treatment initiation and detection of tumor metastases. Cases experiencing non-tumor-related death were censored from survival analysis. The cases that died or euthanized for unknown reasons were counted as events for statistical purposes. The Kaplan-Meier product limit method was used for describing survival characteristics. The log-rank test or the log-rank test for trend were used to assess survival differences between groups. GraphPad Prism 7 (GraphPad Software, Inc., CA, USA) was used for the illustration of Kaplan-Meier survival curves and calculation of the *P*-values in the log-rank test and the log-rank test for trend. The optimal cut-off values of continuous variables were determined by using X-tile plot software version 3.6.1 (Yale University School of Medicine, CT, USA), which assesses the optimal cut-point based on Chi-square values defined by the log-rank test. Univariate and multivariate Cox regression analysis was used for the construction of an integrated prognostic model. The variables that had P-values less than 0.1 in univariate analysis were included in stepwise multivariate model. P-values less than 0.05 were considered to be significant. The P-values were calculated by using JMP software version 12, 64-bit (SAS Institute Inc., Cary, NC, USA).

### Other statistical analysis

JMP Version 12, 64-bit or GraphPad Prism 7 was used for each statistical analysis. For single comparisons, a parametric test (two-tailed unpaired Student’s t-test) and a nonparametric test (Mann-Whitney U test) were used for comparing the means and medians of two groups, respectively. For multiple comparisons, a parametric test (one-way analysis of variance, ANOVA) and a non-parametric test (Kruskal-Wallis test) were first used for examining whether the samples originated from the same distribution. Then, a parametric test (the Turkey-Klemer test) and a non-parametric test (the Steel-Dwass test) were performed as the post-hoc tests for comparing several groups. The cluster analysis, dendrogram, and heatmap were illustrated using JMP software version 12, 64-bit. The clusters were determined by Ward’s method. Multivariate correlation analysis was performed to examine the correlation between miRNA levels and the other parameters using JMP Version 12, 64-bit. Calculated correlations were summarized to a correlation map by using Cytoscape (Version 3.4.0., Cytoscape Consortium, https://cytoscape.org/). For further assessment of the correlation between 2 variables, a scatter plot was constructed and linear regression analysis was performed by using GraphPad Prism 7 (GraphPad Software, Inc.). A *P*-value of less than 0.05 was considered significant.

## Additional file

Additional file 1: **Figure S1:** Study design and the cohort characteristics (age and weight). **Figure S2:** Survival difference between chemotherapeutic regimens. **Figure S3:** Normalization of circulating miR-214 and -126 levels in the clinical samples. **Figure S4:** Circulating miR-214 and -126 levels in the samples that are experimentally hemolyzed and anticoagulated with EDTA and SC. **Figure S5:** Modified -∆CT values and the distance of best cut-off points from the mean. **Figure S6:** Profiles of circulating miR-214 and -126 in appendicular osteosarcoma. **Figure S7: ** Independent profiles of circulating miR-214 and -126 in appendicular osteosarcoma. **Figure S8:** Association of circulating miR-214 and -126 levels with histopathologic indices and subtypes. **Figure S9:** Multivariate correlation analysis and map. (PDF 1160 kb)

## Abbreviations

ACRQ: Adjusted-calibrated relative quantities
ALP: Alkaline phosphatase
cDNA: complementary deoxyribonucleic acid
CRQ: Calibrated relative quantities
Ct: Threshold cycle
DFS: Disease-free survival
EDTA: Ethylenediaminetetraacetic acid
H&E: Hematoxylin and eosin
KM curves: Kaplan-Meier curves
miR-126: microRNA-126
miR-214: microRNA-214
miRNA: microRNA
MST: Median survival time
OS: Overall survival
OSA: Osteosarcoma
RNA: Ribonucleic acid
RQ: Relative quantities
RT-qPCR: Reverse transcription quantitative polymerase chain reaction
SC: Sodium citrate
SD: Standard deviation
