# Supplementary material for: Prognostic significance of circulating microRNA-214 and -126 in dogs with appendicular osteosarcoma receiving amputation and chemotherapy
Source: BMC Vet Res. 2019 Jan 25;15:39. doi: 10.1186/s12917-019-1776-1 (PMC6347759; doi:10.1186/s12917-019-1776-1)
Supplement: Supplementary file 1 — Figure S1: Study design and the cohort characteristics (age and weight). Figure S2: Survival difference between chemotherapeutic regimens. Figure S3: Normalization of circulating miR-214 and -126 levels in the clinical samples. Figure S4: Circulating miR-214 and -126 levels in the samples that are experimentally hemolyzed and anticoagulated with EDTA and SC. Figure S5: Modified -∆CT values and the distance of best cut-off points from the mean. Figure S6: Profiles of circulating miR-214 and -126 in appendicular osteosarcoma. Figure S7: Independent profiles of circulating miR-214 and -126 in appendicular osteosarcoma. Figure S8: Association of circulating miR-214 and -126 levels with histopathologic indices and subtypes. Figure S9: Multivariate correlation analysis and map. (PDF 1160 kb) [file 12917_2019_1776_MOESM1_ESM.pdf]

Supplemental Materials

Prognostic significance of circulating microRNA-214 and -126 in dogs with appendicular osteosarcoma receiving amputation and chemotherapy

Kazuki Heishima,[1*] Travis Meuten,[2] Kyoko Yoshida,[1] Takashi Mori,[1, 3] Douglas H. Thamm[2]

[1] Laboratory of Veterinary Clinical Oncology, Faculty of Applied Biological Sciences, Gifu University, 1-1 Yanagido, Gifu, Japan
[2] Flint Animal Cancer Center, Department of Clinical Sciences, Colorado State University, Fort Collins, Colorado 80523, USA
[3] Center for Highly Advanced Integration of Nano and Life Sciences (G-CHAIN), Gifu University, 1-1 Yanagido, Gifu, Japan

* Corresponding author: Kazuki Heishima

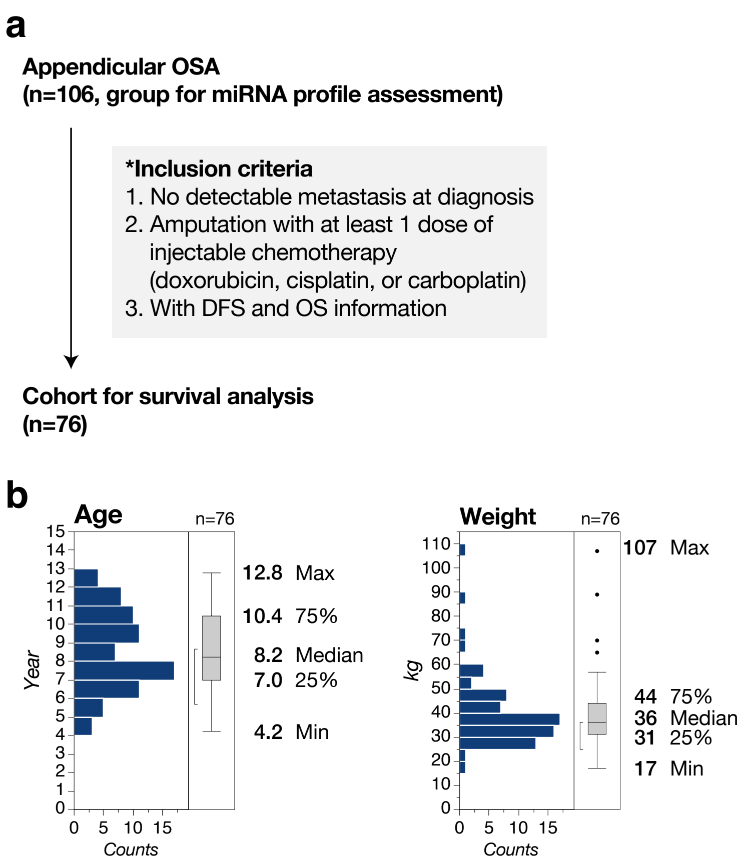

Figure S1. Study design and the cohort characteristics (age and weight)

Appendicular osteosarcoma (OSA, n=106) were collected to assess the profiles of circulating miR-214 and -126. Cases fulfilling all the criteria (n=76) were included in the subsequent survival analysis. The cohort for survival analysis represented typical characteristics of dogs bearing appendicular osteosarcoma. **(a)** Study design, inclusion criteria, and the numbers of the cases included. **(b)** The distribution of age and weight of the cohort for the survival analysis.

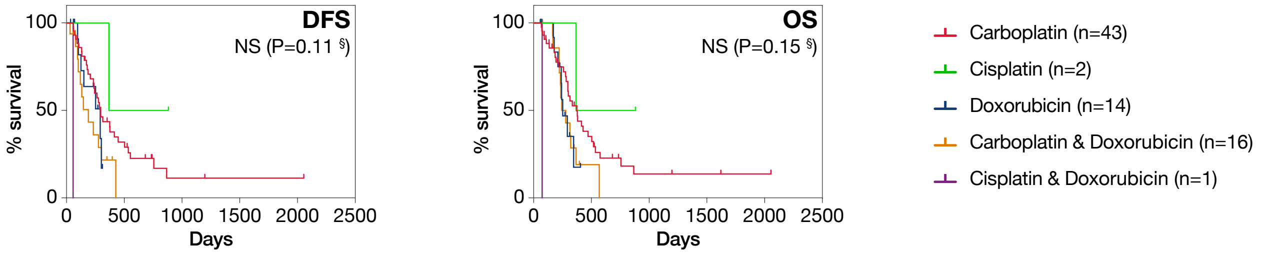

Figure S2. Survival difference between chemotherapeutic regimens

Cases treated with different chemotherapeutic agents showed no significant difference in either disease-free survival (DFS) or overall survival (OS). §: the log-rank test was performed to evaluate survival differences. The cases treated with cisplatin alone (n=2) or combination of cisplatin and doxorubicin (n=1) were not included to the statistical analysis due to insufficient case numbers. A P-value less than 0.05 was considered to be significant.

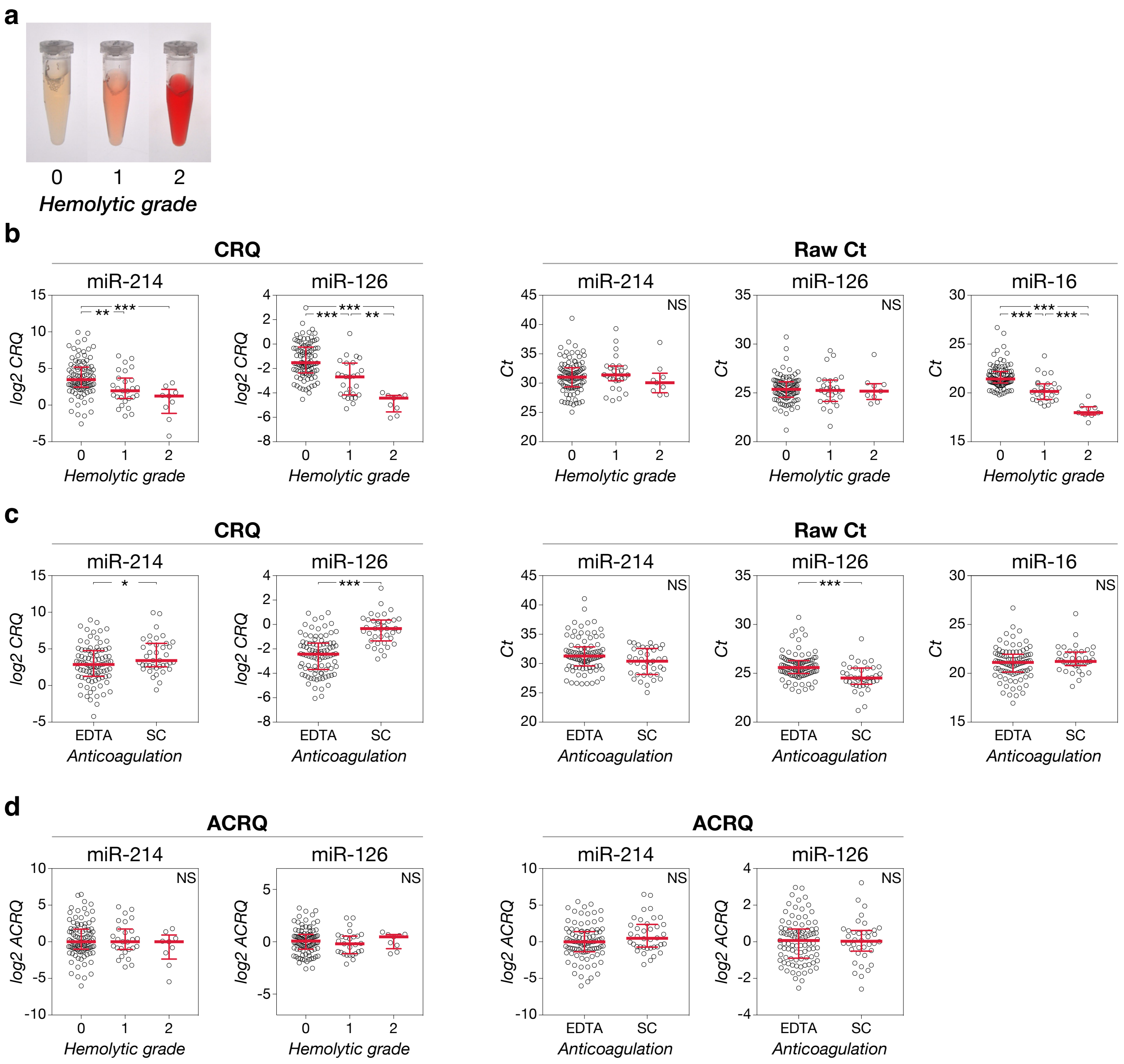

e

P-values	*CRQ* miR-214	*CRQ* miR-126	*Raw Ct* miR-214	*Raw Ct* miR-126	*Raw Ct* miR-16	*ACRQ* miR-214	*ACRQ* miR-126
Hemolytic grade (Kraskal-Wallis)	<0.0001 ***	<0.0001 ***	0.39	0.89	<0.0001 ***	0.62	0.59
Posthoc (Steel-Dwass)							
0 vs 1	0.0077 **	<0.0001 ***	-	-	<0.0001 ***	-	-
0 vs 2	0.0009 ***	<0.0001 ***	-	-	<0.0001 ***	-	-
1 vs 2	-	0.004 **	-	-	0.0001 ***	-	-
Anticoagulation (Mann-Whitney)	0.026 *	<0.0001 ***	0.059	<0.0001 ***	0.25	0.09	0.8

Figure S3. Normalization of circulating miR-214 and -126 levels in the clinical samples

The biases due to the hemolytic grades and types of anticoagulants were normalized by using the median in each grade or type of anticoagulant. **(a)** examples of hemolytic grades. **(b)** Calibrated relative quantities (CRQ) of circulating miR-214 and -126, and raw Ct values of circulating miR-214, -126, and -16 in each hemolytic grade. **(c)** CRQ of circulating miR-214 and -126, and raw Ct values of circulating miR-214, -126, and -16 in each type of anticoagulant. **(d)** Adjusted CRQ (ACRQ) of circulating miR-214 and -126 in each hemolytic grade or type of anticoagulant. **(e)** Summary of statistical analysis and P-values performed in this figure. The center bars and error bars indicate the median and the 25-75% interquartile range, respectively. The Mann-Whitney test, Kraskal-Wallis test, and the Steel-Dwass test were performed for the simple comparisons, multiple comparisons, and the post-hoc analysis, respectively. P-values less than 0.05 were considered to be significant.

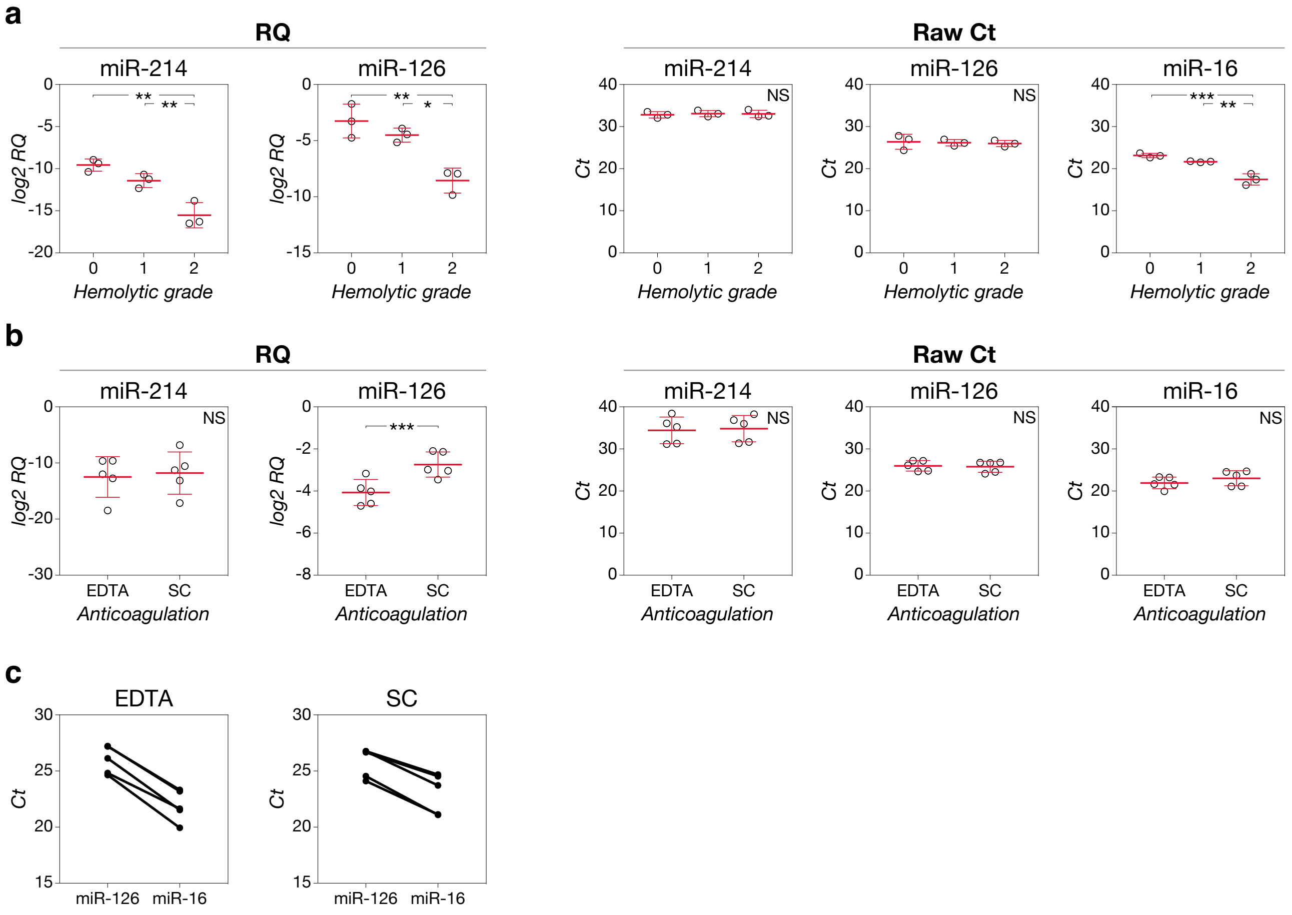

d

P-values	*RQ* miR-214	miR-126	*Raw Ct* miR-214	miR-126	miR-16
Hemolytic grade (ANOVA)	<0.0013 **	<0.003 **	0.9	0.91	0.0004 ***
Posthoc (Tukey)					
0 vs 1	0.17	0.42	-	-	0.15
0 vs 2	0.0012 **	0.003 **	-	-	0.0004 ***
1 vs 2	0.008 **	0.011 *	-	-	0.0019 **
Anticoagulation (t-test)	0.77	0.0086 **	0.85	0.79	0.31

Figure S4. Circulating miR-214 and -126 levels in the samples that are experimentally hemolyzed and anticoagulated with EDTA and SC

The biases observed in the clinical samples were experimentally confirmed. **(a)** Relative quantities (RQ) of circulating miR-214 and -126, and raw Ct values of circulating miR-214, -126, and -16 in each hemolytic grade of experimentally hemolyzed samples. **(b)** RQ of circulating miR-214 and -126, and raw Ct values of circulating miR-214, -126, and -16 in each type of anticoagulant. **(c)** Raw Ct values of miR-126 and -16 in the EDTA and SC samples. The samples that were anticoagulated with EDTA showed higher miR-126 and lower miR-16 than the samples that are anticoagulated with SC. **(d)** Summary of statistical analysis and P-values performed in this figure. The center bars and error bars indicate the median and the 25-75% interquartile range, respectively. The two-tailed Student t-test (unpaired), one-way analysis of variance (ANOVA) test, and the Tukey-Kramer test were performed for the simple comparisons, the multiple comparisons, and the post-hoc analysis, respectively. P-values less than 0.05 were considered to be significant.

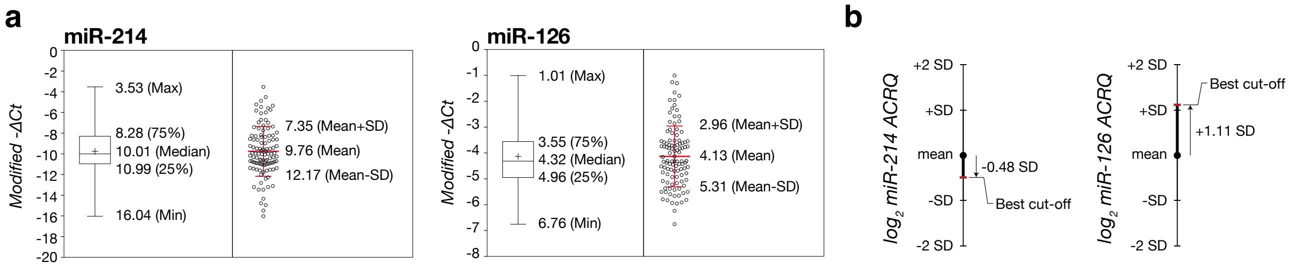

Figure S5. Modified -ΔCT values and the distance of best cut-off points from the mean

(a) Modified -ΔCt values of circulating miR-214 and -126. **(b)** The distance of best cut-off points from the mean. SD, standard deviation.

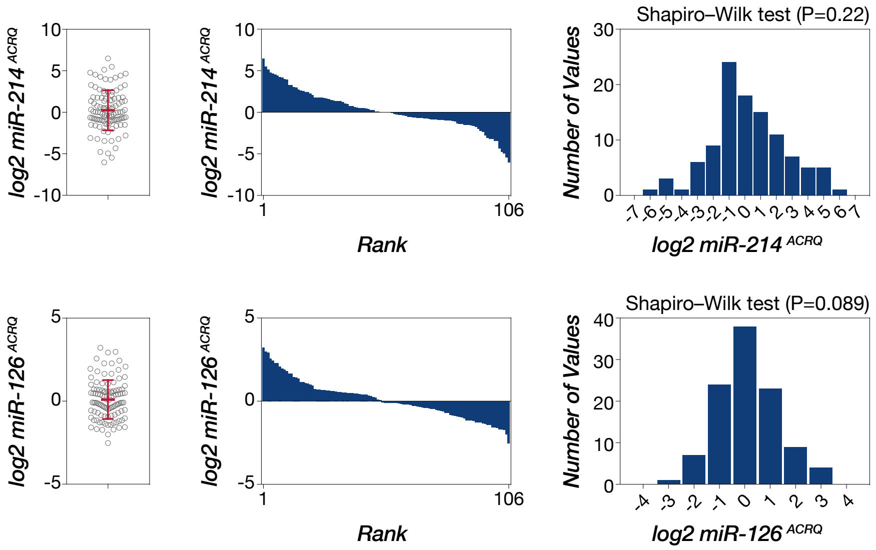

Figure S6. Profiles of circulating miR-214 and -126 in appendicular osteosarcoma

Circulating miR-214 and -126 (log-transformed) showed normal distribution (Shapiro-Wilk test, P=0.22 for circulating miR-214, P=0.089 for circulating miR-126). All values indicated here are log2-transformed adjusted-calibrated relative quantities (ACRQ). P-values less than 0.05 were considered to be significant.

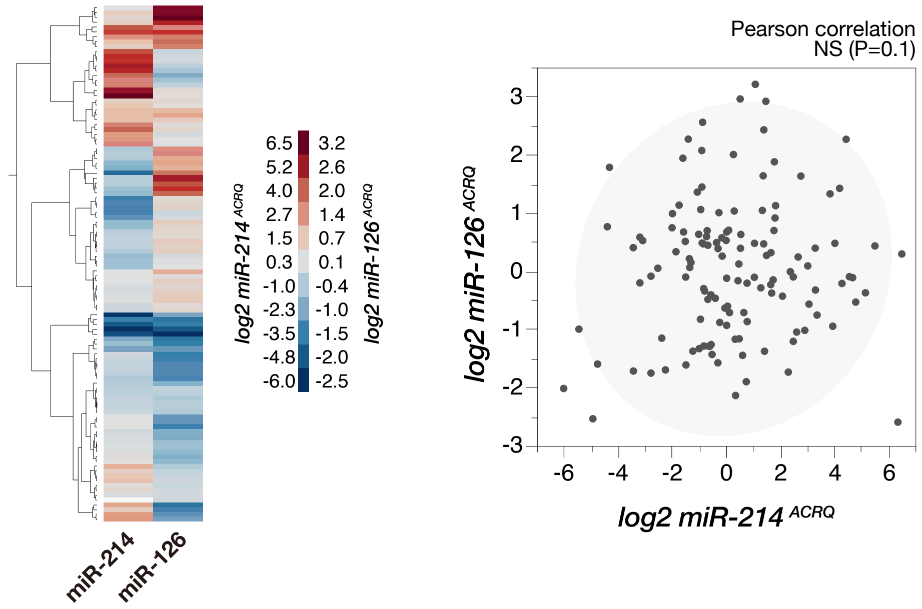

Figure S7. Independent profiles of circulating miR-214 and -126 in appendicular osteosarcoma

Circulating miR-214 and -126 (log-transformed) showed independent profiles. Hierarchical cluster analysis and scatter plot of log-transformed circulating miR-214 and -126 levels. Circulating miR-214 and -126 showed no significant correlation with each other (Pearson's correlation, P=0.1). Ward's method was used for clustering and illustration of the dendrogram. Red and blue in the heatmap indicate high and low levels of circulating miR-214 or -126, respectively. The gray circle in the scatter plot indicates the 95% confidence ellipse. All values indicated here are log2-transformed adjusted-calibrated relative quantities (ACRQ). P-values less than 0.05 were considered to be significant.

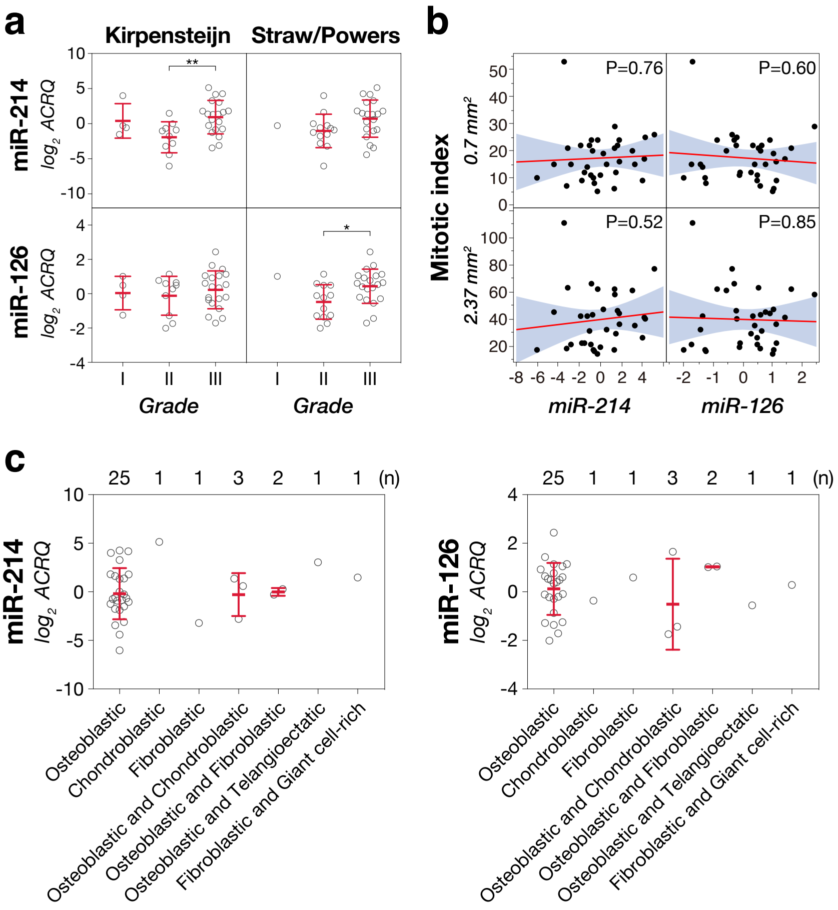

Figure S8. Association of circulating miR-214 and -126 levels with histopathologic indices and subtypes

Circulating miR-214 and -126 showed no association with most histopathological features, although they showed some association with the histological grades. **(a)** Circulating miR-214 and -126 levels of each grade in the Kirpensteijn and the Straw/Powers system. The center bars and error bars indicate the arithmetic mean and standard deviation (SD), respectively. One-way analysis of variance (ANOVA) and the Tukey-Kramer test were performed for the multiple comparisons and the post-hoc analysis, respectively. P-values less than 0.05 were considered significant (*P<0.05, **P<0.01). **(b)** Scatter plots of the mitotic index and the two circulating miRNA levels. Circulating miR-214 and -126 showed no significant correlation with the mitotic indexes that were calculated in 0.7 mm2 (equivalent to 3 high-power fields) and 2.37 mm2 (equivalent to 10 high-power fields). Red lines and blue area indicate the best-fitting lines and the 95% confidential interval of the linear regression, respectively. P-values (two-tailed) were calculated based on the null hypothesis that the overall slope is zero. **(c)** Levels of circulating miR-214 and -126 in each histopathological subtype. The osteoblastic subtype accounted for the majority of the cases, while the population of the other subtypes was small. The center bars and error bars indicate the arithmetic mean and SD, respectively. Only the descriptive analysis was performed due to the small case population included in each group except for the osteoblastic subtype. All circulating miR-214 and -126 values indicated in this figure are log2-transformed adjusted-calibrated relative quantities (ACRQ).

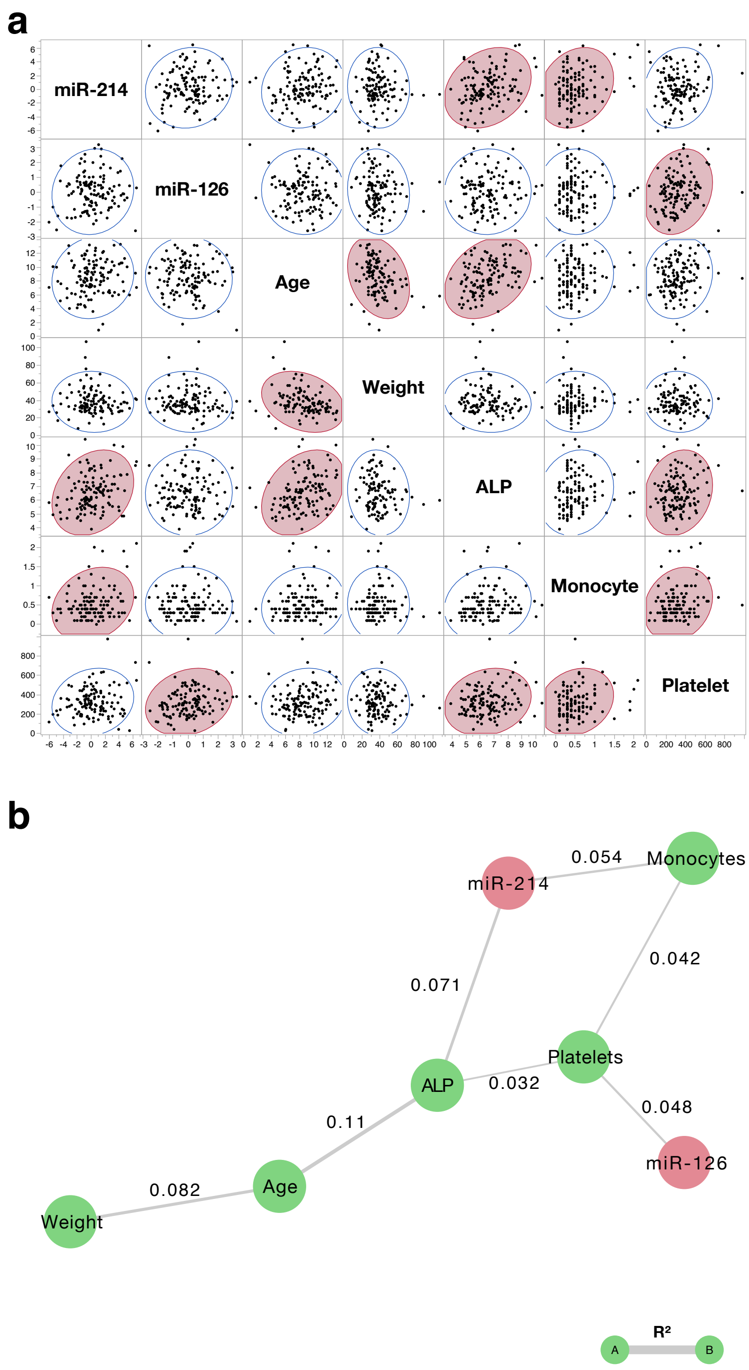

Figure S9. Multivariate correlation analysis and map

Circulating miR-214 and -126 levels displayed no strong correlation with the other clinical parameters. Although several clinical parameters showed significant correlations, the degree of the correlations was weak (R-square values less than 0.2). **(a)** Multivariate correlation analysis. Scatter plots show the correlation between circulating miR-214, -126 levels, age, weight, serum ALP activity, monocyte, and platelet counts. Circles indicate the 95% confidence ellipses. Red and blue circles in the plot indicate significant and non-significant correlation between the two parameters (Pearson's correlation), respectively. One outlier was excluded from the analysis due to the extraordinary high platelet count. **(b)** Correlation map. The correlations between parameters were illustrated as the circle connected to each other with bands. R-square values are displayed on the band between two parameters, and the band thickness represents the degree of correlation. Correlations only with statistical significance were illustrated on the map. P-values less than 0.05 were considered to be significant.
